# Duality and quantum state engineering in cavity arrays

**DOI:** 10.1038/s41598-017-08569-8

**Published:** 2017-08-23

**Authors:** Nilakantha Meher, S. Sivakumar, Prasanta K. Panigrahi

**Affiliations:** 1 Materials Science Group, Indira Gandhi Centre For Atomic Research, Homi Bhabha National Institute, Kalpakkam, 603102 Tamilnadu India; 2 Indian Institute of Science Education and Research Kolkata, Mohanpur, 741246 West Bengal India

## Abstract

A system of two coupled cavities with *N* − 1 photons is shown to be dynamically equivalent to an array of *N* coupled cavities containing one photon. Every transition in the two cavity system has a dual phenomenon in terms of photon transport in the cavity array. This duality is employed to arrive at the required coupling strengths and nonlinearities in the cavity array so that controlled photon transfer is possible between any two cavities. This transfer of photons between two of the cavities in the array is effected without populating the other cavities. The condition for perfect transport enables perfect state transfer between any two cavities in the array. Further, possibility of high fidelity generation of generalized NOON states in two coupled cavities, which are dual to the Bell states of the photon in the cavity array, is established.

## Introduction

Quantum theory provides for fundamentally newer ways of realizing secure communication, faster computation and precision metrology. Quantum networks are basic to implementing these ideas. Physical systems such as spin chains, cavity arrays, Josephson junction arrays, quantum dots, etc., have been investigated to explore their potential for network implementation.

Coupled-cavity arrays have been used extensively in generating nonclassical states of the electromagnetic field and quantum information processing^1, 2^. The technology has matured to such an extent that precise control of the cavity field dynamics is possible. Suitable tailoring of inter-cavity coupling has been put to use in entanglement generation^3^, quantum state preparation^4, 5^, state transfer between spatially separated cavities^6, 7^, exhibition of quantum interference^8, 9^, Rabi oscillation^10^, to mention a few. Consideration of cavities filled with nonlinear media has led to exploration of other quantum phenomena^11^, such as photon blockade^12–14^ and localization-delocalization related to bunching and antibunching of photons^8, 15, 16^. Interplay between intra-cavity nonlinearity and inter-cavity coupling strength has been exploited to control the photon statistics in cavity^17^. Engineering of coupling in arrays of cavities, spins and Josephson junctions offers the possibility of simulating the effects of disorder^18^, phase transitions^19–23^, etc. in condensed matter physics.

The identical and indistinguishable nature of photons require that the number of photons and the number of levels to be occupied by them are considered together. For example, on the consideration of blackbody radiation, Planck distribution is obtained for *N* photons to be distributed in *g* levels when the number of possible ways of distributing as (*N* + *g* − 1)!/*N*!(*g* − 1)!. It is interesting to note that the result is the same if there are *g* − 1 photons and *N* + 1 levels. This possibility of interchanging the roles of the number of particles and the number of levels is a duality. Another simple example of duality is in Euler characteristic *V* − *E* + *F* = 2, where *V*, *E* and *F* refer to the number of vertices, edges and faces respectively of a convex solid. In this expression the roles of *V* and *F* are interchangeable. Such duality relationships are very much sought after in physics, which often facilitates understanding of nontrivial aspects of one system in terms of easily accessible features of the other^24^. In this report, a duality is established between two dynamical systems, namely, one photon in an array of *N* cavities and *N* − 1 photons in two coupled cavities, considering both linear and Kerr-nonlinear cavities. Every transition in the two cavity system has a dual phenomenon in terms of photon transport in the cavity array. This feature helps to identify the conditions required in the linear cavity array for a perfect transport of photon between two cavities equidistant from the respective ends. The result is generalized to nonlinear cavities which allows perfect transport between any two cavities in the array. Another prospect that makes this study interesting in the context of information transfer is the possibility of perfect state transfer from one cavity to another. Our results also point to the possibility of generating NOON states, which have found diverse applications and relate to a Bell state of the dual system^25–28^.

## Model and Analysis

Consider a system of two linearly coupled cavities described by the Hamiltonian

1 $${H}_{A}={\omega }_{1}{a}_{1}^{\dagger }{a}_{1}+{\omega }_{2}{a}_{2}^{\dagger }{a}_{2}+J[{a}_{1}^{\dagger }{a}_{2}+{a}_{1}{a}_{2}^{\dagger }].$$

Here *ω*_1_ and *ω*_2_ are the resonance frequencies of the respective cavities and *J* is the coupling strength. Suffix *A* has been used to refer to this system of two coupled cavities. Here *a*_1(2)_ and ${a}_{\mathrm{1(2)}}^{\dagger }$ are the annihilation and creation operators for the first(respectively, second) cavity. Let |*n* + 1〉 represent the bipartite state |*N* − 1 − *n*, *n*〉 of the two cavities corresponding to *N* − *n* − 1 quanta in the first cavity and *n* quanta in the second cavity. The total number of quanta in the two cavities is *N* − 1. If the number of photons is fixed to be *N* − 1, the Hamiltonian is expressed as

2 $${H}_{A}=\sum _{n=0}^{N-1}{{\rm{\Omega }}}_{n+1}|n+1\rangle \langle n+1|+\sum _{n=0}^{N-2}{J}_{n+1}(|n+1\rangle \langle n+2|+|n+2\rangle \langle n+1|),$$

where Ω_*n*+1_ = [(*N* − 1 − *n*)*ω*_1_ + *nω*_2_] and ${J}_{n+1}=\sqrt{(n+1)(N-1-n)}J$.

Now consider a system of *N* linearly coupled cavities, described by

3 $${H}_{B}=\sum _{l=1}^{N}{\tilde{\omega }}_{l}{b}_{l}^{\dagger }{b}_{l}+\sum _{l=1}^{N-1}{\tilde{J}}_{l}({b}_{l}^{\dagger }{b}_{l+1}+{b}_{l}{b}_{l+1}^{\dagger }),$$

where ${\tilde{\omega }}_{l}$ is the cavity resonance frequency for the *l*th cavity, *b*_*l*_ and ${b}_{l}^{\dagger }$ the annihilation and creation operators for the *l*th cavity. The strength of coupling between the *l* and (*l* + 1) cavities in the array is ${\tilde{J}}_{l}$. This form of the Hamiltonian can be mapped to that of a spin network which has been studied in the context of state transfer and entanglement generation^29^. For a single quantum in the system, the possible states are |*l*〉〉, which represents one photon in the *l*th cavity while the other cavities are in their respective vacuua. Then the Hamiltonian is

4 $${H}_{B}=\sum _{l=1}^{N}{\tilde{\omega }}_{l}|l\rangle \rangle \langle \langle l|+\sum _{l=1}^{N-1}{\tilde{J}}_{l}(|l\rangle \rangle \langle \langle l+1|+|l+1\rangle \rangle \langle \langle l|).$$

Duality of the two systems described by *H*_*A*_ and *H*_*B*_ respectively is identified if ${\tilde{J}}_{l}=\sqrt{l(N-l)}J$, ${\tilde{\omega }}_{l}=[(N-l){\omega }_{1}+(l-\mathrm{1)}{\omega }_{2}]$ and *l* = *n* + 1. The transition $|N-1-n,n\rangle \to |N-2-n,n+1\rangle$ in the system of two cavities corresponds to photon transport from |*n* + 1〉〉 → |*n* + 2〉〉 in the array. In essence, transitions in the two-cavity system are equivalent to transport of a photon across the cavities in the array.

If the initial state of the two cavity system at resonance (Δ = *ω*_1_ − *ω*_2_ = 0) is *N*-n-1, n > it evolves to

5 $$\begin{array}{ccc}{e}^{-i{H}_{A}t}|N-1-n,n\rangle & = & {e}^{-i(N-1)\omega t}\sum _{k=0}^{N-1-n}\sum _{l=0}^{n}(\begin{array}{c}N-1-n\\ k\end{array})(\begin{array}{c}n\\ l\end{array}){(\cos Jt)}^{N-1-(k+l)}{(-i\sin Jt)}^{k+l}\\  &  & \times \sqrt{(\begin{array}{c}N-1\\ n\end{array})/(\begin{array}{c}N-1\\ n+k-l\end{array})}|N-1-(n+k-l),n+k-l\rangle,\end{array}$$

at time *t*. It is worth noting that the time-evolved state is an atomic coherent state^30^.

**Figure 1:**
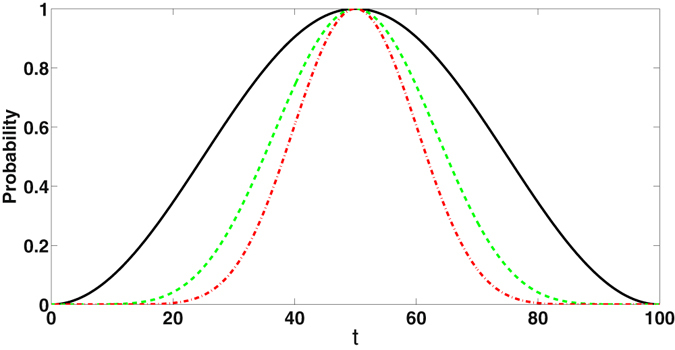
Time evolution of probability for the coupled cavity to be in $|0,N-1\rangle$ on evolution from the initial state $|N-1,0\rangle$, with *J* = 10^−2^ *π*. By duality, these profiles show the probability of detecting a single photon in *N*th (end) cavity in the cavity array. Different curves correspond to *N* = 2 (continuous), 4 (dashed)and 6 (dot-dashed).

At *t* = *π*/2*J* the time-evolved state is $|n,N-1-n\rangle$, corresponding to swapping the number of photons in the cavities. Time evolution of the respective probabilities for $|N-\mathrm{1,0}\rangle$ to become $|0,N-1\rangle$ corresponding to *N* = 2, 4 and 6 are shown in Fig. 1. Complete transfer of photons between the end cavities of the array corresponds to |*N* − 1, 0〉 → |0, *N* − 1〉 transition in the coupled cavity system. By the duality between *H*_*A*_ and *H*_*B*_, these profiles also represent the probability of transferring a photon from one end to the other in an array of 2, 4 and 6 cavities respectively. It may be noted that the probabilities attain their peak value of unity, corresponding to complete transport of a quantum between the end cavities, when *t* = *π*/2*J*.

It is essential that *J*_*l*_ are related to *J* in the specified manner. It is of interest to note that the requirement for such inhomogeneous couplings in linear quantum spin networks  and optical waveguide arrays has been explored^31, 32^. If the coupling strengths *J*_*l*_ are equal and all the cavities are identical, the average number of quanta at time *t* in the *j*-th cavity is given by

6 $${\langle {n}_{j}\rangle }_{t}={\langle {b}_{j}^{\dagger }{b}_{j}\rangle }_{t}=\sum _{l=1}^{N}{|{G}_{jl}|}^{2}{\langle {b}_{l}^{\dagger }{b}_{l}\rangle }_{0}.$$

where,

7 $${G}_{jl}=\sqrt{(\frac{2}{N+1})}\sum _{k=1}^{N}{e}^{-i(\tilde{\omega }+2J\cos (\frac{\pi k}{N+1}))t}\,\sin (\frac{j\pi k}{N+1})\sin (\frac{l\pi k}{N+1}).$$

**Figure 2:**
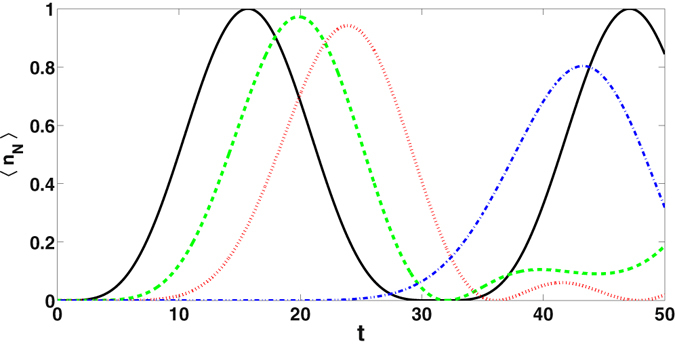
Average number of photon in the end cavity as a function of t in cavity array. Number of cavities in the array is *N* = 3 (solid line), 4 (dashed), 5 (dotted) and 10 (dot-dashed). All the cavities are identical and homogeneously coupled with coupling strength $J=\sqrt{2}\mathrm{/10}$. The system parameters are normalized in the unit of $\tilde{\omega }$.

Here $\tilde{\omega }$ is the resonance frequency of the cavities in the array. If the quantum is initially in the first cavity, that is, ${\langle {b}_{l}^{\dagger }{b}_{l}\rangle }_{0}={\delta }_{\mathrm{1,}l}$, then

8 $${\langle {n}_{N}\rangle }_{t}={\langle {b}_{N}^{\dagger }{b}_{N}\rangle }_{t}={|{G}_{N1}|}^{2},$$

is the average photon number in the last cavity. For large *N*, sin(*Nkπ*/*N* + 1) ≈ sin(*kπ*) = 0 and *G*_*N*1_ tends to zero. This indicates that complete transfer is not possible. Time evolution of the average number of quantum 〈*n*_*N*_〉 in the end cavity for arrays with *N* = 3, 4, 5 and 10 cavities respectively are shown in Fig. 2. From the figure, it is clear that complete transfer occurs if the array has three cavities. Maximum of |*G*_*N*1_|^2^ decreases with increasing the number of cavities. Hence, complete transfer does not occur if the homogeneously coupled array has more than three cavities whereas inhomogeneous coupling achieves complete transfer in shorter time^31, 33^.

It is to be further noted that complete transition is possible only between the states $|N-1-n,n\rangle$ and $|n,N-1-n\rangle$ of the coupled cavities. Analogously complete transfer of a single photon can occur only in between (*n* + 1)th and (*N* − *n*)th cavities in the cavity array. With linear coupling, it is not possible to achieve complete transfer between two arbitrary cavities in the array.

To see if nonlinearity helps in steering the evolution of states to achieve perfect transfer, we consider the Kerr-type nonlinearity. We present the analysis of two coupled nonlinear cavities. The required Hamiltonian is

9 $${H}_{A}^{^{\prime} }={\omega }_{1}{a}_{1}^{\dagger }{a}_{1}+{\omega }_{2}{a}_{2}^{\dagger }{a}_{2}+{\chi }_{1}{a}_{1}^{\dagger 2}{a}_{1}^{2}+{\chi }_{2}{a}_{2}^{\dagger 2}{a}_{2}^{2}+J[{a}_{1}^{\dagger }{a}_{2}+{a}_{1}{a}_{2}^{\dagger }],$$

which describes two linearly coupled Kerr cavities.

If it is required to evolve from |*m*, *n*〉 to |*p*, *q*〉, consider the superposition $|{X}_{\pm }\rangle =\mathrm{1/}\sqrt{2}(|m,n\rangle \pm |p,q\rangle )$. These two states become approximate eigenstates of the ${H}_{A}^{^{\prime} }$ if *J* ≪ *χ*_1_, *χ*_2_, *ω*_1_, *ω*_2_, and

10 $${\rm{\Delta }}=\frac{(p(p-\mathrm{1)}-m(m-\mathrm{1))}{\chi }_{1}+(q(q-\mathrm{1)}-n(n-\mathrm{1))}{\chi }_{2}}{m-p}.$$

This condition is equivalent to

11 $$\langle m,n|{H}_{A}^{^{\prime} }|m,n\rangle =\langle p,q|{H}_{A}^{^{\prime} }|p,q\rangle .$$

This equality of average energy in the two states is another way of stating the requirement that the states |X±⟩ are approximate eigenstates of ${H}_{A}^{^{\prime} }$. In the discussion that follows it is assumed that *χ*_1_ = *χ*_2_ = *χ* > 0 and the condition simplifies to Δ = 2*χ*(*n* − *p*).

If the initial state is $|m,n\rangle = 1/\sqrt{2}(|{X}_{+}\rangle + |{X}_{-}\rangle)$, the state of the system at a later time is, $|\psi (t)\rangle \approx$ $[\cos ({\theta }_{a}t)|m,n\rangle -i\,\sin ({\theta }_{a}t)|p,q\rangle ]$, with ${\theta }_{a}=({\lambda }_{s}^{a}-{\lambda }_{n}^{a}\mathrm{)/2}$. Here ${\lambda }_{s}^{a}$ and ${\lambda }_{n}^{a}$ are the eigenvalues of ${H}_{A}^{^{\prime} }$ corresponding to the approximate eigenvectors |*X*_+_〉 and |*X*_−_〉 respectively. At $t=\pi /({\lambda }_{s}^{a}-{\lambda }_{n}^{a})$, the time-evolved state is |*p*, *q*〉. This is the minimum time required to switch from |*m*, *n*〉 to |*p*, *q*〉. Thus, the state switching (SS) condition given in Eq. (10) ensures that there is complete transfer from the initial state |*m*, *n*〉 to the desired final state |*p*, *q*〉.

**Figure 3:**
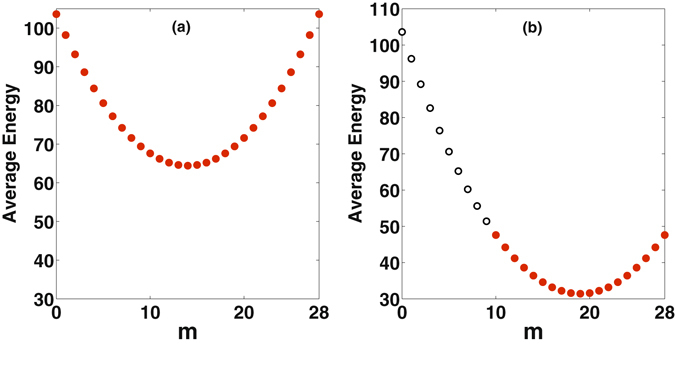
Average energy $\langle m,n|{H}_{A}^{^{\prime} }|m,n\rangle$ as a function of *m*, with Δ = 0, *χ* = 0.1 (Left) and Δ = −2, *χ* = 0.1 (Right). The system parameters are expressed in units of *ω*_1_.

It is immediate that detuning Δ and nonlinear coupling strength *χ* can be properly chosen for a given value for *n* − *p*. As the value of *n* is specified in the initial state |*m*, *n*〉, the two parameters Δ and *χ* fix the number of quanta say *s*, that can be transferred and the target state becomes $|p=m\pm s,q=n\mp s\rangle$. It needs to be emphasized that for a given Δ and *χ* satisfying the SS condition, no more than two states can have their average energies equal as shown in Fig. 3. Once these parameters are fixed, probability of transition to any state other than the target state is negligible. Hence, Δ and *χ* provide control to steer the system from the initial state |*m*, *n*〉 to the final state |*p*, *q*〉.

In Fig. 3, $\langle m,n|{H}_{A}^{^{\prime} }|m,n\rangle$ is plotted as a function of *m*, keeping *m* + *n* = 28 fixed. From Fig. 3(a), it is seen that every state has only one partner state with equal average energy. So, SS can occur between these partner states. It is observed from Fig. 3(b) that not every state has a partner state with equal average energy. Essentially, states without partner states are approximate eigenstates of *H*′_*A*_ and, therefore, do not evolve. This brings out another control aspect available in the system, namely, the possibility of inhibiting evolution of certain states with properly chosen values of the control parameters *χ* and Δ.

**Figure 4:**
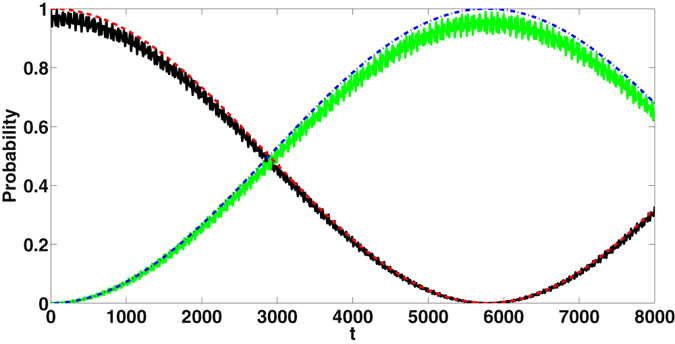
Probability of detecting the state |14〉 from |50〉 as a function of *t*. Detecting other states are practically zero. Continuous black(dashed) and continuous green (dashed-dot) line corresponds to *P*_50_ and *P*_14_ calculated numerically (approximate analytical solution $|\psi (t)\rangle$). We set Δ = −0.2, *χ* = 0.1, *J* = 0.035. The system parameters are used in the unit of *ω*_1_.

Consider the initial state of the coupled cavity system to be $|50\rangle$. In Fig. 4, the probability of detecting the system in the state $|14\rangle$ at later times is shown when the required SS condition is satisfied. The values have been generated from the approximate evolved state |ϕ(t)〉 and also by exact numerical solution of the evolution corresponding to ${H}_{A}^{^{\prime} }$. It is seen that the quanta are exchanged periodically driving the system between |14〉 and |50〉 and transfer to other states is insignificant.

**Figure 5:**
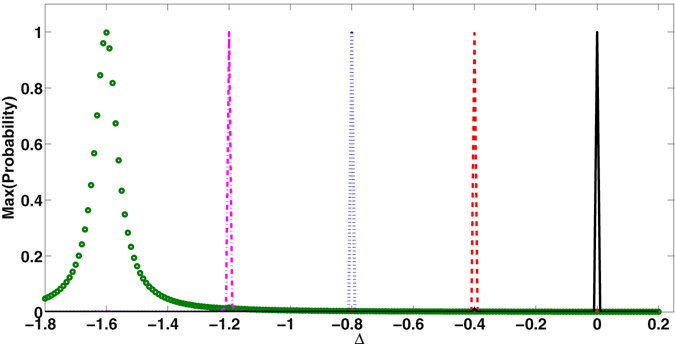
Maximum probability of detecting quantum states |*p*, *q*〉 as a function of Δ for *χ* = 0.2 and *J* = 0.05 from the initial state |5, 0〉. Note that, complete switching occurs from |5, 0〉 to |41〉 (circle), |32〉 (dot-dashed), |23〉 (dotted), |14〉 (dashed) and |05〉 (continuous) at Δ = −8*χ*, Δ = −6*χ*, Δ = −4*χ*, Δ = −2*χ* and Δ = 0 respectively satisfying the relation Eq. (10). The system parameters are expressed in the unit of *ω*_1_.

In order to effect transition to other states, the value of Δ can be chosen properly. The maximum probabilities of detecting the target state $|p,q\rangle$ with *p* = 1, 2, 3, 4, 5 and *p* + *q* = 5 from |50〉 are shown in Fig. 5 as a function of Δ. The value of *χ* has been chosen to be 0.2. Depending on the value of detuning, exchange of quanta is precisely controlled to different target states.

A duality relation of the two cavity system with the cavity array system is possible in this nonlinear case too. Consider the nonlinear cavity array Hamiltonian

12 $${H}_{B}^{^{\prime} }=\sum _{l=1}^{N}{\tilde{\omega }}_{l}{b}_{l}^{\dagger }{b}_{l}+{\tilde{\chi }}_{l}{({b}_{l}^{\dagger }{b}_{l})}^{2}+\sum _{l=1}^{N-1}\sqrt{l(N-l)}J({b}_{l}^{\dagger }{b}_{l+1}+{b}_{l}{b}_{l+1}^{\dagger }),$$

which includes Kerr nonlinearity in each cavity of the array. This is dual to ${H}_{A}^{^{\prime} }$ if ${\tilde{\omega }}_{k+1}+{\tilde{\chi }}_{k+1}=(N-1-k){\omega }_{1}+$ $k{\omega }_{2}+[(N-1-k)(N-2-k)+k(k-\mathrm{1)]}\chi$. With this identification, transitions among levels in the two Kerr cavity system can be mapped to transfer of photon in the Kerr cavity array.

In particular, transition from $|N-1-n,n\rangle$ to $|N-1-q,q\rangle$ in the coupled cavities corresponds to transferring a photon between (*n* + 1)-cavity to (*q* + 1)-cavity in the cavity array. The condition to realize this transfer is $\langle \langle n+1|{H}_{B}^{^{\prime} }|n+1\rangle \rangle =\langle \langle q+1|{H}_{B}^{^{\prime} }|q+1\rangle \rangle$, whose dual relation for the coupled cavities is given in Eq. (11). For the Hamiltonian ${H}_{B}^{^{\prime} }$, this condition yields,

13 $${\tilde{\chi }}_{k+1}+{\tilde{\omega }}_{k+1}=(N-1){\omega }_{1}-2k\chi (n+q+1-N)+[(N-1-k)(N-2-k)+k(k-1)]\chi $$

to realize complete transfer of photon occurs between the cavities. On employing cavity-dependent nonlinearity ${\tilde{\chi }}_{l}$, controlled transfer of photons between selected cavities is achievable. Such site-dependent nonlinearity has been realized recently by embedding quantum dots in each photonic crystal cavities^34–36^.

In the limit of weak coupling strength *J*, $\frac{1}{\sqrt{2}}(|n+1\rangle \rangle \pm |q+1\rangle \rangle )$ are eigenstates of ${H}_{B}^{^{\prime} }$ and the corresponding eigenvalues are denoted by ${\lambda }_{s}^{b}$ and ${\lambda }_{n}^{b}$. The initial state $|n+1\rangle \rangle$ evolves under ${H}_{B}^{^{\prime} }$ to $|\psi (t)\rangle \rangle \approx \,\cos ({\theta }_{b}t)|$ $n+1\rangle \rangle -i\,\sin ({\theta }_{b}t)|q+1\rangle \rangle ,$ where ${\theta }_{b}=({\lambda }_{s}^{b}-{\lambda }_{n}^{b}\mathrm{)/2}$. It is seen that the photon is exchanged periodically between the cavities. An important feature of this process is that the other cavities in the array are not populated to any appreciable extent during the evolution. This conclusion is based on the observation that the states other than $|n+1\rangle \rangle$ and $|q+1\rangle \rangle$ do not contribute appreciably to $|\psi (t)\rangle \rangle$.

If the coupling term in the Hamiltonian ${H}_{A}^{^{\prime} }$ is taken to be $J[{e}^{i\eta }{a}_{1}^{\dagger }{a}_{2}+{e}^{-i\eta }{a}_{1}{a}_{2}^{\dagger }]$ making the coupling constants complex, then the initial state |*m*, *n*〉, evolves to $|\psi (t)\rangle \approx [{\rm{c}}{\rm{o}}{\rm{s}}({\theta }_{a}t)|m,n\rangle -i{e}^{-i(q-n)\eta }{\rm{s}}{\rm{i}}{\rm{n}}({\theta }_{a}t)|p,q\rangle ]$. These states are of the form $|\psi \rangle ={\rm{c}}{\rm{o}}{\rm{s}}\theta |m,n\rangle +{e}^{i\varphi }{\rm{s}}{\rm{i}}{\rm{n}}\theta |p,q\rangle$, if *θ* = *θ*_*a*_ *t* and *ϕ* = −(*π*/2 + (*q* − *n*)*η*).

If *m* ≠ *p* and *θ* ≠ 0, ±*π*/2, then $|\psi \rangle$ is entangled. Additionally, if *m*, *q* = *N* and *θ* = *π*/4, the resultant state is

$$|\psi \rangle =\frac{1}{\sqrt{2}}(|N0\rangle +{e}^{i\varphi }|0N\rangle ),$$

the generalized NOON state. In the case of cavity array this is equivalent to generating the Bell state $|\psi \rangle \rangle ={\rm{c}}{\rm{o}}{\rm{s}}\theta |n+1\rangle \rangle +{e}^{i\varphi }{\rm{s}}{\rm{i}}{\rm{n}}\theta |q+1\rangle \rangle$.

Another important outcome of the complex coupling in the context of single photon in cavity array is the possibility of state transfer between *any* two cavities. Consider the initial state of the cavity array to be $\alpha |{\rm{vac}}\rangle \rangle +\beta |n+1\rangle \rangle$, which corresponds to the (*n* + 1)-th cavity in the superposition *α*|0〉 + *β*|1〉 and the other cavities are in their respective vacuua. If the SS condition is satisfied, the time-evolved state is $\alpha |{\rm{vac}}\rangle \rangle +\beta {e}^{-i\lambda t}({\rm{c}}{\rm{o}}{\rm{s}}{\theta }_{b}t|n+1\rangle \rangle -i{e}^{-i\eta (q-n)}{\rm{s}}{\rm{i}}{\rm{n}}{\theta }_{b}t|q+1\rangle \rangle )$, where $\lambda =({\lambda }_{s}^{b}+{\lambda }_{n}^{b}\mathrm{)/2}$. At 2*θ*_*b*_ *t* = *π*, the state of the (*q* + 1)-th cavity is the superposition *α*|0〉 + *β*|1〉 and the other cavities in their respective vacuua for the suitable value of *η*. Thus, the SS condition ensures the state of the field in the (*n* + 1)-th cavity is transferred to the (*q* + 1)-th cavity.

It is possible to implement the above scheme for photon transport and state transfer in arrays of high quality photonic crystal cavities (PCC)^37^. Typical values for the cavity resonance frequencies of PCC are in the range of mega-Hertz to tera-Hertz. The Q values of PCC are of the order of ~10^6^ with low modal volume^38^. High value of Q implies that the dissipation is less. Kerr nonlinearity in PCC is realized by embedding two level atoms (quantum dots) in the cavities with the advantage that the realizable nonlinearity is much larger compared to the optical nonlinearities in solids^39^. Using these typical values, the effect of dissipation on the photon transport and state transfer is shown to be negligible. For the purpose of demonstration, an array of six identical cavities, whose resonant frequency is 62.5 THz, has been considered. The Kerr nonlinearity parameters ${\tilde{\chi }}_{l}$ are determined using the relation given in Eq. (13) for *χ* = 1.25 THz and *ω*_1_ = 12.5 THz. The coupling strength is chosen to be *J* = 70 GHz, which is easily achievable in PCC^40^. The effect of dissipation is quantified by the fidelity between the state realized at the target cavity (penultimate cavity in this study) in the presence and absence of dissipation. The state of the target cavity has been determined by numerically solving the Lindblad evolution equation and the estimated fidelity is 0.98. This clearly shows that the choice of the inter-cavity couplings and Kerr nonlinearities given by the duality principle is robust enough to achieve near perfect transfer with the currently available technology. Similar results are possible with other platforms such as the Josephson junction arrays^41–43^.

## Summary

Dynamics of a single photon transport in an array of *N* cavities is dual to the problem of sharing of (*N* − 1) photons between two coupled cavities. This duality is extendable even if the cavities are of Kerr-type, which, in turn, requires the couplings to be inhomogeneous. Duality between the two systems makes it transparent to identify the correct combination of the coupling strengths and local nonlinearities in the array for complete photon transfer between any two cavities. In the linear case, perfect transport is possible only between the cavities which are symmetrically located from the end cavities of the array. With Kerr nonlinear cavities, perfect transport between any two cavities, without any restriction whatsoever, is possible. Importantly, this transfer is effected without populating the other cavities in the array, so that the transfer cannot be viewed as a continuous hopping of photon from one cavity to the other. Another interesting result of the analysis is the possibility of perfect transfer of states of the form $\alpha |0\rangle +\beta |1\rangle$, achieved by a combination of Kerr nonlinearity and complex coupling strengths among the cavities in the array. This feature is important in the context of encoding and transfer of information. Further, high fidelity generation of entangled states $\cos \,\theta |m,n\rangle +{e}^{i\varphi }\,\sin \,\theta |p,q\rangle$ is possible in the coupled Kerr nonlinear cavities provided the conditions for state switching are satisfied. By duality, this implies that Bell states $\cos \,\theta |10\rangle +{e}^{i\varphi }\,\sin \,\theta |01\rangle$ are realizable with high fidelity on account of perfect state transfer in the cavity array, which is the dual process corresponding to state switching in the coupled cavities. These results are pertinent in the context of quantum information processing using cavity arrays as they are scalable. The ideas presented here are applicable to coupled spin chains as well to achieve controlled transfer of states between any two spins in the chain.
